# Association between sleep duration and dyslipidemia in premenopausal and postmenopausal women

**DOI:** 10.1186/s12889-026-27011-1

**Published:** 2026-03-17

**Authors:** Miyeong Jung, Sung-il Cho

**Affiliations:** 1 School of Public Health, Seoul National University, Seoul, Republic of Korea; 2 Seoul National University Graduate School of Public Health, 1 Gwanak-ro, Gwanak-gu, Seoul, 08826 Korea

**Keywords:** Sleep Duration, Dyslipidemia, Triglyceride, Menopause, Postmenopause

## Abstract

**Background:**

Cardiovascular disease (CVD) is one of the leading causes of death worldwide, and dyslipidemia—a major risk factor for CVD—varies by sex, age, and menopausal status. While some studies have explored the relationship between sleep duration and dyslipidemia, findings remain inconsistent, and most have not accounted for menopausal status. This study investigates the association between sleep duration and dyslipidemia in premenopausal and postmenopausal women.

**Methods:**

Data from the 2014–2022 Korea National Health and Nutrition Examination Survey (KNHANES) were used to examine 10,115 women aged 40–64 years (4,428 premenopausal; 5,687 postmenopausal). Sleep duration, obtained through self-report, was classified into four groups: <6 h, 6-<7 h, 7-<8 h (reference), and ≥ 8 h per day. Multivariable logistic regression models were applied to evaluate the associations between sleep duration and dyslipidemia, as well as individual lipid abnormalities. Analyses were further conducted separately according to menopausal status.

**Results:**

Sleep duration was significantly associated with hypertriglyceridemia. Using 7-<8 h of sleep as the reference category, Model 1 showed higher odds of hypertriglyceridemia among individuals sleeping < 6 h (odds ratio [OR]: 1.68; 95% confidence interval [CI]: 1.34–2.10) and ≥ 8 h (OR: 1.40; 95% CI: 1.15–1.71). In Model 2, adjusted for all covariates, these associations remained significant for < 6 h (OR: 1.42; 95% CI: 1.13–1.79) and ≥ 8 h (OR: 1.33; 95% CI: 1.09–1.64). In subgroup analyses, among premenopausal women, sleeping ≥ 8 h was associated with higher odds of dyslipidemia compared with the reference group (7-<8 h) (OR: 1.22; 95% CI: 1.02–1.46) and hypo-HDL-cholesterolemia (OR: 1.29; 95% CI: 1.06–1.57). Among postmenopausal women, both short (< 6 h; OR: 1.35; 95% CI: 1.02–1.80) and long sleep duration (≥ 8 h; OR: 1.36; 95% CI: 1.05–1.77) were associated with higher odds of hypertriglyceridemia. No statistically significant associations were observed for hyper-LDL-cholesterolemia.

**Conclusions:**

Sleep duration showed a U-shaped association with hypertriglyceridemia in both premenopausal and postmenopausal women.

**Supplementary Information:**

The online version contains supplementary material available at 10.1186/s12889-026-27011-1.

## Background

 Cardiovascular disease (CVD) continues to be among the top causes of mortality around the world [1]. Among modifiable risk factors, major contributors include hypertension, diabetes, dyslipidemia, and smoking [2]. Dyslipidemia, characterized by abnormal plasma lipid levels—such as elevated triglyceride (TG), increased low-density lipoprotein cholesterol (LDL-C), or reduced high-density lipoprotein cholesterol (HDL-C)—is a key factor in CVD development [3]. Despite its clinical significance, dyslipidemia is often underdiagnosed and inadequately managed, contributing to a rising global burden [4].

Serum lipid concentrations vary by age and sex, with notable differences before and after menopause [5]. Menopause induces changes in lipid metabolism [6], yet research specifically examining dyslipidemia risk factors in premenopausal and postmenopausal women remains limited.

Sleep duration is another crucial determinant of metabolic health, with the National Sleep Foundation recommending 7 to 9 hours of sleep per night for adults [7]. Women, particularly those undergoing menopause, frequently experience sleep disturbances that may impact lipid metabolism [8].

Lifestyle-based interventions, such as improving diet, increasing physical activity, quitting smoking, and limiting alcohol consumption, play a central role in dyslipidemia management. Studies have demonstrated that behavioral modifications can significantly reduce dyslipidemia incidence and severity [9]. Although some research has explored associations between sleep duration and lipid profiles [10–12], findings have been inconsistent, and studies focusing specifically on premenopausal and postmenopausal women remain scarce.

Given the marked differences in dyslipidemia and serum lipid levels across age and sex—particularly before and after menopause—this study aims to examine the relationship between sleep duration, which is often affected by menopausal transition, and dyslipidemia among premenopausal and postmenopausal women.

## Methods

### Study design and population

Data from the 2014–2022 Korea National Health and Nutrition Examination Survey (KNHANES) were analyzed in this cross-sectional study. KNHANES is a nationally representative survey designed to assess the health and nutrition status of the Korean population, collecting comprehensive information on health behaviors, lifestyle factors, and biochemical measurements, including blood lipid profiles. The survey was conducted triennially from Phase I (1998) to Phase III (2005) and was subsequently restructured into a continuous annual survey system beginning with Phase IV (2007–2009), which has been maintained to the present.

Participants were selected from KNHANES cycles VI, VII, and VIII, with inclusion restricted to individuals surveyed between 2014 and 2022. Since Phase IV, KNHANES has operated as a continuous, nationally representative survey employing a complex, multistage probability sampling framework. Although cycle VI included data from 2013 to 2015, the present study limited the analytic period to 2014 onward to maintain consistency in physical activity assessment, a potential confounding factor in the relationship between sleep duration and lipid outcomes. Beginning in 2014, KNHANES implemented the Global Physical Activity Questionnaire (GPAQ), replacing the previously used short-form International Physical Activity Questionnaire (IPAQ), which improved comparability of physical activity measures across survey cycles [13]. The most recent dataset used in this analysis, corresponding to the 2022 survey year, became publicly available in January 2024. The KNHANES protocol was reviewed and approved by the Institutional Review Board of the Korea Disease Control and Prevention Agency, and all participants provided written informed consent [13]. The current study was granted exemption status by the Institutional Review Board of Seoul National University (IRB No. SNU 24-09-077).

**Fig. 1:**
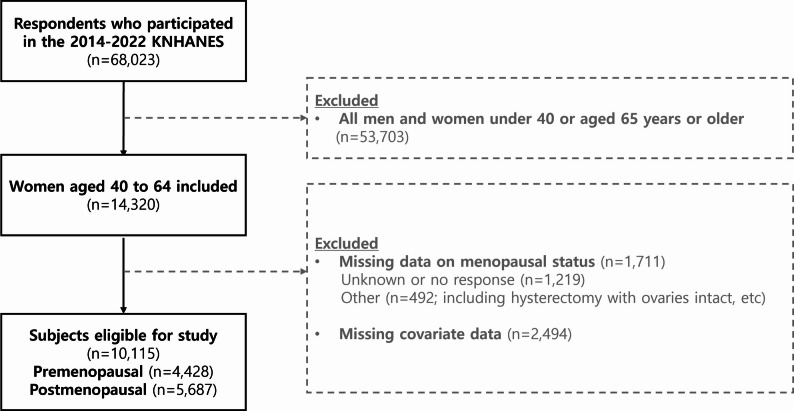
Flowchart of the selection process

Data from women aged 40–64 who took part in the 2014–2022 KNHANES survey was used for the study population. Based on prior research [14], participants who responded to the question about menstrual status with ‘currently menstruating’, ‘pregnant’, or ‘postpartum and breastfeeding’ were designated as premenopausal. Participants reporting either ‘natural menopause’ or ‘surgical menopause’ were classified as postmenopausal. Among the 68,023 individuals surveyed between 2014 and 2022, only those with complete data were included in the final analysis, with incomplete records excluded using a complete-case approach. For the final analysis, we selected 4,428 premenopausal women and 5,687 postmenopausal women based on relevant health interview and examination items, and a flowchart of the study subjects eligible for this research is presented in Fig. 1.

### Measures

#### Sleep duration

Sleep duration, the main exposure variable, was derived from responses to self-reported sleep related questions in the KNHANES data. The difference between wake-up time and bedtime was determined to estimate the average daily sleep duration. When participants reported different sleep durations for weekends and weekdays, average sleep duration per day was derived using the following method, based on a prior method [15]$:\left(\right(weekdaysleepduration\times5days)+(weekendsleepduration\times2days\left)\right)/7.$ The result was rounded to one decimal place. The calculated average daily sleep duration was then categorized into four groups for analysis: <6, 6-<7, 7-<8, and ≥ 8 h per day, with 7-<8 h used as the reference group.

#### Dyslipidemia and serum lipid profiles

Serum lipid profiles, including TG, LDL-C, and HDL-C, were measured using venous serum samples from study participants who fasted for at least 8 hours. LDL-C levels were estimated using the Friedewald formula when triglyceride levels were < 200 mg/dL, and directly measured LDL-C values provided by KNHANES were used for participants with triglyceride levels ≥ 200 mg/dL to avoid potential inaccuracies of the Friedewald formula at higher triglyceride concentrations [16]. In this study population, approximately 90% of participants had triglyceride levels < 200 mg/dL and had LDL-C estimated using the Friedewald formula, while about 10% had triglyceride levels ≥ 200 mg/dL and were assigned directly measured LDL-C values. Hypertriglyceridemia was defined as fasting TG concentration ≥ 200 mg/dL. Hyper-LDL-cholesterolemia was defined as a fasting LDL-C concentration ≥ 160 mg/dL or current use of lipid-lowering medication. Hypo-HDL-cholesterolemia was defined as an HDL-C < 50 mg/dL for women. These biochemical cut-points were based on established criteria used in national dyslipidemia surveillance and prior studies [3]. For descriptive purposes, we also identified participants who reported a prior physician diagnosis of dyslipidemia, to account for individuals whose lipid levels may have been normalized due to ongoing lipid-lowering treatment at the time of the survey. In the main analysis, dyslipidemia was treated as a binary outcome defined by the presence of any of the above biochemical lipid abnormalities, and each lipid abnormality was additionally analyzed as a separate outcome.

#### Covariates

Based on previous studies [3, 9, 12], covariates were selected as factors that may influence sleep duration or dyslipidemia. These included demographic factors (age, economic activity status, income level, and education level), clinical factors (hypertension and diabetes), and lifestyle factors, including smoking status, alcohol intake, physical activity, body mass index [BMI].

For general characteristics variables, demographic factors (age, economic activity status, income level, and education level) were self-reported using self-administered questionnaires [17]. Economic activity status was classified as either employed or unemployed (non-economically active population) based on responses to a question regarding employment status. Income level was classified into four groups, from low to high, based on household income quartiles. Education level was divided into four categories, ranging from less than elementary school graduate to college graduate or higher.

All covariates, other than age and socioeconomic status, reflect health-related characteristics. As clinical factors, hypertension and diabetes were considered present if participants reported a doctor’s diagnosis, as per the health survey on disease prevalence. As lifestyle factors, BMI was measured using a standardized procedure, with body weight (in kilograms) divided by height (in meters) squared to calculate the value. BMI was classified into three groups: underweight/normal weight (< 23 kg/m^2,^ due to small sample sizes, these categories were combined), overweight (23.0–24.9 kg/m^2^), and obese (≥ 25.0 kg/m^2^). Smoking status was assessed into two groups: current smokers and those who had either quit smoking or never smoked (due to small sample sizes, these categories were combined). Drinking status was categorized based on alcohol intake frequency in the past year: those who drank at least once a month and those who drank less than once a month. Finally, to assess physical activity, the aerobic physical activity compliance indicator provided by KNHANES was used. According to this indicator, participants were divided into two groups: an aerobic physical activity group and an inactivity group based on the ‘aerobic physical activity participation rate’ provided by the KNHANES, measured through the GPAQ. Participants were classified as aerobically active if they reported at least 150 min of moderate-intensity activity per week or 75 min of vigorous-intensity activity, with vigorous activity weighted as double the duration of moderate activity. Those who did not meet these thresholds were categorized as inactive.

### Statistical analysis

A final analytic sample of 10,115 participants was selected after excluding individuals with missing data and responses of ‘don’t know’ or ‘refused to answer’. A complex survey design with appropriate sample weights was applied to account for the multistage stratified sampling method used in KNHANES.

Weighted descriptive statistics were computed using weighted chi-square tests and t-tests to compare menopausal groups. Multivariate logistic regression models were used to examine the association between sleep duration and dyslipidemia, with relevant covariates included in the models. To examine the association between specific serum lipid profiles and sleep duration, lipid abnormalities were categorized as hypertriglyceridemia, hyper-LDL-cholesterolemia, and hypo-HDL-cholesterolemia, following established definitions. Odds ratios (ORs) with 95% confidence intervals (CIs) were estimated for each sleep duration category, using 7-<8 hours as the reference group. This category was selected because it corresponded to the median sleep duration in the study population and represented the central tendency of the distribution, providing a statistically stable and epidemiologically appropriate reference for comparison. Stratified analyses by menopausal status were conducted to estimate the predicted odds ratios for each lipid profile component, and results were visualized graphically. In supplementary analyses, sleep duration was additionally modeled as a continuous variable using linear and quadratic terms to explore potential functional forms of association. Additional analyses were performed to evaluate the stability of the results after excluding implausible sleep duration values (< 3 or > 14 h/day) and trimming the upper and lower 1% of triglyceride values. These supplementary analyses were performed using the same covariate adjustments as in the fully adjusted models (Model 2), and the results are presented in the Supplementary Information.

To formally evaluate whether associations between sleep duration and lipid outcomes differed by menopausal status, interaction terms between sleep duration and menopausal status were included in pooled multivariable models and tested using design-based Wald tests.

To account for multiple comparisons in analyses involving multiple lipid outcomes, false discovery rate (FDR) correction was applied to the sleep duration coefficients in the fully adjusted models (Model 2) across lipid outcomes using the Benjamini–Hochberg procedure [18]. FDR correction was applied to the primary hypothesis tests in the main fully adjusted models, while stratified analyses were considered exploratory and were not subjected to multiplicity adjustment. Statistical significance was defined as a two-sided p-value < 0.05. All statistical analyses were conducted using R software (version 4.3.3).

## Results

### Characteristics of Study population

After selecting the eligible subjects, 10,115 participants were included in one or more analyses. Table 1 provides an overview of study participant characteristics by menopausal status (premenopausal vs. postmenopausal). The mean age of the study population was 52.2 ± 7.2 years, with 4,428 (43.8%) premenopausal and 5,687 (56.2%) postmenopausal women. Among postmenopausal women, menopause type was further classified as natural or surgical, and natural menopause accounted for 89.1% of postmenopausal cases. The mean sleep duration was 7.0 ± 1.3 h per day overall, with premenopausal women reporting a slightly longer sleep duration (7.1 ± 1.2 h) than postmenopausal women (6.8 ± 1.3 h). Short sleep duration (< 6 h) was more prevalent among postmenopausal women than premenopausal women (15.3% vs. 11.1%). Conversely, more premenopausal women reported sleeping 8 or more hours compared to postmenopausal women (30.7% vs. 26.7%).

**Table 1:** General Characteristics of the Study population by Menopausal status (*n* = 10,115)

Characteristic	Total *N* = 10,115	Premenopausal *N* = 4,428 (43.8%)	Postmenopausal *N* = 5,687 (56.2%)	*p*-value^†^	
	*N* (%) or mean ± SD	*N* (%) or mean ± SD	*N* (%) or mean ± SD		
*Menopause type*					
Natural menopause			5,068 (89.1)		
Surgical menopause			619 (10.9)		
*Age (years)*	52.2 ± 7.2	45.6 ± 3.9	57.4 ± 4.5	**< 0.001**	
*Economic activity*				**< 0.001**	
Employed	5,935 (58.3)	2,816 (62.9)	3,119 (54.3)		
Unemployed	4,180 (41.7)	1,612 (37.1)	2,568 (45.7)		
*Income quartiles*				**< 0.001**	
Lowest	1,078 (9.8)	300 (6.5)	778 (12.7)		
Lower middle	2,427 (23.5)	966 (22.0)	1,461 (24.8)		
Upper middle	3,046 (30.8)	1,449 (33.2)	1,597 (28.6)		
Highest	3,564 (36.0)	1,713 (38.3)	1,851 (33.9)		
*Education*				**< 0.001**	
Below elementary school graduate	1,266 (10.8)	84 (1.9)	1,182 (18.6)		
Middle school graduate	1,212 (11.0)	168 (3.9)	1,044 (17.3)		
High school graduate	4,067 (42.1)	1,849 (42.4)	2,218 (41.8)		
College graduate or higher	3,570 (36.1)	2,327 (51.8)	1,243 (22.3)		
*Hypertension*				**< 0.001**	
No	8,362 (83.6)	4,106 (92.6)	4,256 (75.6)		
Yes	1,753 (16.4)	322 (7.4)	1,431 (24.4)		
*Diabetes mellitus*				**< 0.001**	
No	9,485 (94.2)	4,327 (97.7)	5,158 (91.1)		
Yes	630 (5.8)	101 (2.3)	529 (8.9)		
*Body mass index (BMI)*				**< 0.001**	
Under and normal (BMI < 23)	5,538 (55.4)	2,663 (59.8)	2,875 (51.5)		
Overweight (BMI 23 ~ 25)	2,486 (24.0)	941 (21.5)	1,545 (26.2)		
Obese (BMI ≥ 25)	2,091 (20.6)	824 (18.7)	1,267 (22.4)		
*Physical activity*				**0.004**	
Inactivity	5,648 (54.9)	2,380 (53.2)	3,268 (56.5)		
Aerobic physical activity	4,467 (45.1)	2,048 (46.8)	2,419 (43.5)		
*Smoking*				0.314	
Never or past smoker	9,675 (95.6)	4,219 (95.4)	5,456 (95.8)		
Current	440 (4.4)	209 (4.6)	231 (4.2)		
*Drinking*				**< 0.001**	
At least once a month	5,885 (57.6)	2,193 (50.4)	3,692 (64.1)		
Less than once a month	4,230 (42.4)	2,235 (49.6)	1,995 (35.9)		
*Sleep duration (hours)*	7.0 ± 1.3	7.1 ± 1.2	6.8 ± 1.3	**< 0.001**	
<6	1,352 (13.3)	476 (11.1)	876 (15.3)	**< 0.001**	
6-<7	2,670 (26.2)	1,124 (25.2)	1,546 (27.2)		
7-<8	3,226 (31.9)	1,480 (33.0)	1,746 (30.9)		
≥ 8	2,867 (28.6)	1,348 (30.7)	1,519 (26.7)		
*Dyslipidemia*				**< 0.001**	
No	4,666 (48.0)	2,652 (60.5)	2,014 (36.9)		
Yes	5,449 (52.0)	1,776 (39.5)	3,673 (63.1)		
*Triglyceride (mg/dL)*		116.8 ± 79.1	105.7 ± 72.6	125.4 ± 82.9	**< 0.001**
Normal		9,104 (90.2)	4,096 (92.5)	5,008 (88.2)	**< 0.001**
Hypertriglyceridemia		1,011 (9.8)	332 (7.5)	679 (11.8)	
*LDL-Cholesterol (mg/dL)*	121.8 ± 33.8	118.7 ± 30.6	124.3 ± 35.9	**< 0.001**	
Normal	7,485 (74.9)	3,868 (87.4)	3,617 (63.9)	**< 0.001**	
Hyper-LDL-cholesterolemia	2,630 (25.1)	560 (12.6)	2,070 (36.1)		
*HDL-Cholesterol (mg/dL)*	56.2 ± 13.4	57.4 ± 13.3	55.2 ± 13.4	**< 0.001**	
Normal	6,693 (67.4)	3,136 (71.4)	3,557 (63.9)	**< 0.001**	
Hypo-HDL-cholesterolemia	3,422 (32.6)	1,292 (28.6)	2,130 (36.1)		

Abbreviations: *LDL* low-density lipoprotein, *HDL* high-density lipoprotein, *SD* standard deviation

^**†**^Pearson’s χ^2^ test

Bold values indicate statistical significance (*p*  < 0.05)

### Associations between Sleep duration and Dyslipidemia

Multivariable logistic regression models were used to examine the association between sleep duration and dyslipidemia (Table 2). 

**Table 2:** Associations between Sleep duration and Dyslipidemia by multivariable logistic regression

	Crude	Model 1^a^	Model 2^b^
	OR (95% CI)	OR (95% CI)	OR (95% CI)
Sleep duration(h) (ref. 7-<8)			
<6	1.30 (1.13-1.50)	1.20 (1.03-1.39)	1.01 (0.86-1.18)
6-<7	1.09(0.97-1.22)	1.05 (0.93-1.19)	0.99 (0.87-1.12)
≥8	1.13(1.01-1.27)	1.16 (1.03-1.31)	1.12 (0.99-1.27)
Menopause (ref. pre-menopause)			
Post-menopause		2.62 (2.38-2.89)	1.43 (1.21-1.68)
Age (years)			1.02 (1.01-1.04)
Economic activity (ref. employed)			
Unemployed			1.22 (1.11-1.35)
Income quartiles (ref. Lowest)			
Lower middle			0.98 (0.81-1.19)
Upper middle			0.89 (0.74-1.07)
Highest			0.88 (0.73-1.06)
Education (ref. below elementary school)			
Middle school graduate			0.96 (0.77-1.19)
High school graduate			0.75 (0.63-0.90)
College graduate or higher			0.64 (0.53-0.78)
Hypertension (ref. No)			
Yes			1.76 (1.50-2.05)
Diabetes mellitus (ref. No)			
Yes			4.26 (3.16-5.73)
Smoking (ref. past, non-smoker)			
Current			1.25 (0.99-1.60)
Drinking (ref. less than once a month)			
More than once a month			0.67 (0.61-0.75)
Physical activity (ref. inactivity)			
physical activity			0.92 (0.83-1.01)
Body Mass Index (ref. BMI < 23)			
Overweight (BMI 23~25)			1.93 (1.72-2.17)
Obese (BMI>=25)			2.41 (2.11-2.76)

Footnote: *OR* Odds Ratio, *CI* Confidence Interval

Crude model is unadjusted model

^a^Model 1 adjusted for menopausal status

^b^Model 2 additionally adjusted for age, economic activity, income quartiles, education level, hypertension, diabetes mellitus, smoking, drinking, physical activity, and BMI

Bold values indicate statistical significance (*p* < 0.05)

No correction for multiple comparisons was applied to Table 2, as it presents a single primary outcome model

In the unadjusted (crude) model, both short (<6 hours; OR: 1.30; 95% CI: 1.13-1.50) and long (≥8 hours; OR: 1.13; 95% CI: 1.01-1.27) sleep duration were associated with higher odds of dyslipidemia compared with the reference group (7-<8 hours).

After adjustment for menopausal status (Model 1), these associations remained significant (<6 hours: OR: 1.20; 95% CI: 1.03-1.39; ≥8 hours: OR: 1.16; 95% CI: 1.03-1.31). Postmenopausal women also had substantially higher odds of dyslipidemia than premenopausal women (OR: 2.62; 95% CI: 2.38-2.89). 

However, after full adjustment for sociodemographic, lifestyle, and clinical covariates (Model 2), sleep duration was no longer significantly associated with dyslipidemia. Dyslipidemia remained significantly associated with postmenopausal status, older age, unemployment, hypertension, diabetes mellitus, and obesity, whereas higher education and regular alcohol consumption were associated with lower odds of dyslipidemia. Formal interaction testing did not demonstrate statistically significant effect modification by menopausal status for overall dyslipidemia (p-value for interaction = 0.48).

Overall, 5,449 participants (52.0%) had dyslipidemia, and its prevalence was greater among postmenopausal women than premenopausal women (63.1% vs. 39.5%).

Postmenopausal women were older and had higher TG and LDL-C levels but lower HDL-C levels than premenopausal women. Additionally, they had significantly higher proportions of unemployment, lowest/lower middle-income quartiles, below middle school education, hypertension, diabetes mellitus, overweight/obesity, physical inactivity, regular alcohol consumption (at least once a month), hypertriglyceridemia, hyper-LDL-cholesterolemia, and hypo-HDL-cholesterolemia.

### Associations between Sleep duration and Serum lipid profiles

Logistic regression models were used to examine associations between sleep duration and individual lipid abnormalities (Table 3). 

**Table 3:** Associations between Sleep duration and Serum lipid profiles by multivariable logistic regression

	Hyper-triglyceridemia ^a^			Hyper-LDL-cholesterolemia ^b^			Hypo-HDL-cholesterolemia ^c^		
	Crude	Model 1^d^	Model 2^e^	Crude	Model 1	Model 2	Crude	Model 1	Model 2
Sleep duration(h) (ref. 7-<8)									
<6	1.75(1.40-2.19)	1.68 (1.34-2.10)	1.42 (1.13-1.79)	1.20(1.01-1.41)	1.07 (0.90-1.27)	0.94 (0.79-1.12)	1.15(0.99-1.34)	1.12 (0.96-1.30)	0.99 (0.84-1.16)
6-<7	1.17(0.96-1.42)	1.15 (0.95-1.40)	1.10 (0.90-1.34)	1.04(0.91-1.19)	1.00 (0.87-1.14)	0.93 (0.80-1.07)	1.02(0.90-1.15)	1.01 (0.89-1.14)	0.97 (0.85-1.11)
≥8	1.39(1.14-1.69)	1.40 (1.15-1.71)	1.33 (1.09-1.64)	0.97(0.85-1.11)	0.99 (0.87-1.14)	0.95 (0.83-1.10)	1.10(0.97-1.24)	1.11 (0.98-1.25)	1.06 (0.94-1.21)
Menopause (ref. pre-menopause)									
Post-menopause		1.64 (1.40-1.91)	1.14 (0.86-1.52)		3.91 (3.45-4.43)	1.69 (1.39-2.07)		1.41 (1.28-1.56)	1.10 (0.92-1.30)
Age (years)			1.01 (0.99-1.03)			1.06 (1.05-1.08)			0.99 (0.98-1.00)
Body Mass Index (ref. BMI < 23)									
Overweight (BMI 23~25)			2.32 (1.92-2.79)			1.59 (1.40-1.82)			1.76 (1.56-1.98)
Obese (BMI>=25)			2.69 (2.22-3.28)			1.87 (1.63-2.16)			2.08 (1.83-2.36)
Economic activity (ref. employed)									
Unemployed			1.23 (1.06-1.44)			1.18 (1.05-1.31)			1.10 (1.00-1.22)
Income quartiles (ref. Lowest)									
Lower middle			1.02 (0.80-1.32)			1.20 (0.98-1.48)			1.01 (0.84-1.21)
Upper middle			0.74 (0.57-0.95)			1.14 (0.93-1.39)			0.96 (0.80-1.15)
Highest			0.87 (0.67-1.14)			1.18 (0.96-1.45)			0.94 (0.78-1.13)
Education (ref. below elementary)									
Middle school graduate			1.07 (0.81-1.41)			1.08 (0.87-1.34)			0.86 (0.71-1.04)
High school graduate			0.81 (0.64-1.02)			1.22 (1.02-1.47)			0.72 (0.61-0.85)
College graduate or higher			0.70 (0.53-0.93)			1.25 (1.01-1.54)			0.57 (0.47-0.69)
Hypertension (ref. No)									
Yes			1.14 (0.93-1.40)			1.87 (1.63-2.15)			1.21 (1.05-1.39)
Diabetes mellitus (ref. No)									
Yes			1.57 (1.19-2.07)			2.59 (2.09-3.19)			2.00 (1.62-2.46)
Smoking (ref. past, non-smoker)									
Current			2.24 (1.64-3.07)			1.19 (0.89-1.58)			1.14 (0.90-1.44)
Drinking (ref. less than once a month)									
more than once a month			0.93 (0.79-1.09)			0.86 (0.77-0.97)			0.59 (0.53-0.65)
Aerobic physical activity (ref. inactivity)									
Aerobic physical activity			0.86 (0.74-1.01)			0.99 (0.88-1.10)			0.85 (0.77-0.94)

Footnote: OR = Odds Ratio; CI = Confidence Interval

^a^TG ≥ 200mg/dL

^b^LDL-C ≥ 160mg/dL or use of a lipid-lowering drug

^c^HDL-C < 50mg/dL

Crude model is unadjusted model

^d^Model 1 adjusted for menopausal status

^e^Model 2 additionally adjusted for age, economic activity, income quartiles, education level, hypertension, diabetes mellitus, smoking, drinking, physical activity, and BMI

Bold values indicate FDR-adjusted p-value<0.05 (Benjamini-Hochberg) for sleep duration coefficients in Model 2 only

In the crude model, both short (<6 hours) and long (≥8 hours) sleep duration were associated with higher odds of hypertriglyceridemia, and short sleep was also associated with higher odds of hyper-LDL-cholesterolemia. No significant association was observed between sleep duration and hypo-HDL-cholesterolemia. 

After adjustment for menopausal status (Model 1), sleep duration remained significantly associated only with hypertriglyceridemia. Both short (<6 hours; OR: 1.68; 95% CI: 1.34-2.10) and long (≥8 hours; OR: 1.40; 95% CI: 1.15-1.71) sleep duration were associated with increased odds of hypertriglyceridemia. Menopausal status was significantly associated with all lipid abnormalities in this model. 

After full adjustment (Model 2), the association between sleep duration and hypertriglyceridemia remained significant (<6 hours: OR: 1.42; 95% CI: 1.13-1.79; ≥8 hours: OR: 1.33; 95% CI: 1.09-1.64), whereas menopausal status remained significantly associated only with hyper-LDL-cholesterolemia. Hypertriglyceridemia was independently associated with BMI, socioeconomic indicators, diabetes mellitus, and smoking. Hyper-LDL-cholesterolemia was associated with age, BMI, economic activity, education level, hypertension, diabetes mellitus, and alcohol consumption, while hypo-HDL-cholesterolemia was associated with BMI, education, hypertension, diabetes mellitus, alcohol consumption, and physical activity.

Interaction testing indicated no significant interaction between sleep duration and menopausal status for hypertriglyceridemia or hyper-LDL-cholesterolemia but indicated a significant interaction for hypo-HDL-cholesterolemia (p-value for interaction = 0.044). 

#### Associations between Sleep duration and Dyslipidemia components stratified by Menopausal status

**Fig. 2:**
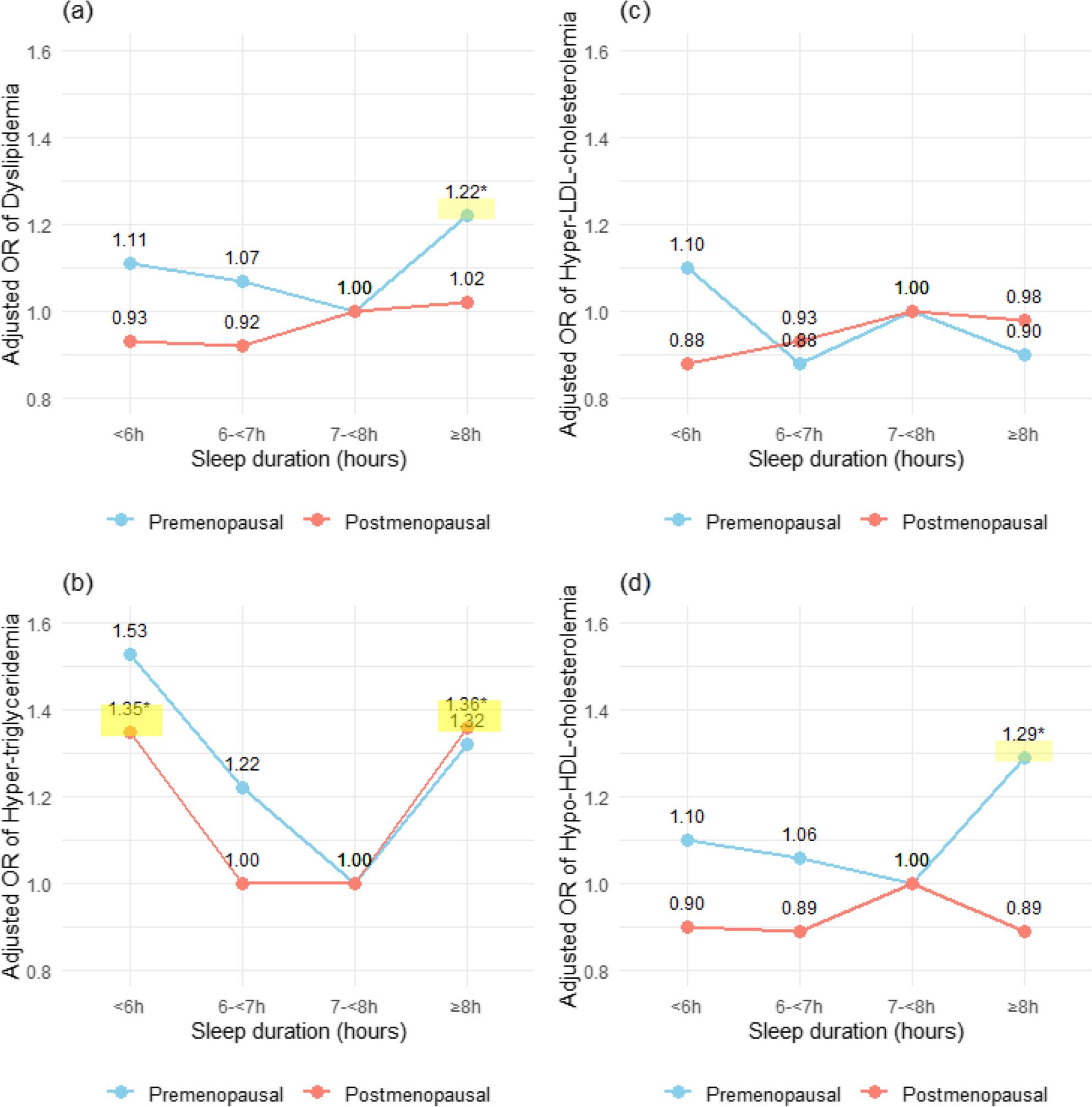
Associations between sleep duration and dyslipidemia components stratified by menopausal status. The graphs show adjusted odds ratio (ORs) for (**a**) dyslipidemia, (**b**) hyper-triglyceridemia, (**c**) hyper-LDL-cholesterolemia, and (**d**) hypo-HDL-cholesterolemia across sleep duration categories. The lines represent ORs, and the shaded areas indicate statistical significance (*p*-value < 0.05). Analyses were adjusted for age, economic activity, income quartiles, education level, hypertension, diabetes mellitus, smoking, drinking, physical activity and BMI

Stratified analyses were conducted to explore potential differences in associations by menopausal status (Figure 2). 

Among premenopausal women, long sleep duration (≥8 hours) was associated with higher odds of dyslipidemia compared with the reference category (7-<8 hours; OR: 1.22; 95% CI: 1.02-1.46). Long sleep duration was also increased odds of hypo-HDL-cholesterolemia (OR: 1.29; 95% CI: 1.06-1.57).

Among postmenopausal women, both short sleep duration (<6 hours; OR: 1.35; 95% CI: 1.02-1.80) and long sleep duration (≥8 hours; OR: 1.36; 95% CI: 1.05-1.77) were associated with higher odds of hyper-triglyceridemia. No significant associations were observed between sleep duration and hyper-LDL-cholesterolemia in either menopausal group.

Overall, stratified analyses suggested potential differences in the pattern of sleep-lipid associations according to menopausal status.

## Discussion

In this cross-sectional analysis using data from the 2014–2022 Korea National Health and Nutrition Examination Survey (KNHANES), we examined the associations between sleep duration and dyslipidemia among premenopausal and postmenopausal women aged 40–64 years. Our findings revealed four key results:

1. Sleep duration was significantly associated with hypertriglyceridemia.
2. This association remained consistent after stratification by menopausal status.
3. Menopausal status was independently associated with dyslipidemia and hyper-LDL-cholesterolemia.
4. Sleep duration was not significantly associated with dyslipidemia, hyper-LDL-cholesterolemia, or hypo-HDL-cholesterolemia after full adjustment and correction for multiple comparisons.

Collectively, these findings suggest that sleep duration may play a more prominent role in triglyceride metabolism than in LDL-C or HDL-C regulation, highlighting hypertriglyceridemia as metabolically sensitive lipid fraction associated with sleep behavior.

Our findings align with prior research suggesting that sleep duration is more consistently associated with triglyceride levels than with LDL-C or HDL-C. Previous systematic reviews and meta-analyses have reported no consistent evidence of a significant relationship between sleep duration and dyslipidemia development [19]. Similarly, earlier KNHANES analyses among adults have reported inconsistent associations between sleep duration and dyslipidemia development overall. Similarly, earlier KNHANES analyses among adults aged ≥ 19 years reported that individuals sleeping less than 6 h had a dyslipidemia prevalence 1.156-fold higher than those sleeping 7–9 h after adjusting for covariates. Notably, when serum lipid levels were analyzed as continuous variables, inconsistent results were observed compared to categorical analyses [12]. Prior studies analyzing sleep duration as both continuous and categorical variables have reported inconsistent associations depending on the modeling approach. These findings highlight the complexity of the relationship between sleep duration and lipid metabolism and suggest that clinically meaningful categorical grouping may provide complementary insights. For instance, research analyzing lipid as continuous variables found that TG and HDL-C exhibit non-linear relationships with sleep duration, with short sleep being associated with high TG and low HDL-C levels [20]. Similarly, a study involving middle-aged and elderly Taiwanese individuals reported a U-shaped relationship between sleep duration and HDL-C levels, where those sleeping less than 6 h or more than 7 h had a higher prevalence of low HDL-C levels compared to those sleeping 6-<7 h [10]. Together, these findings suggest that the relationship between sleep duration and lipid metabolism may be non-linear and lipid-specific.

Menopause represents a critical metabolic transition characterized by declining estrogen levels, redistribution of body fat, and worsening insulin sensitivity, all of which contribute to increased LDL-C and decreased HDL-C [21–23]. Consistent with previous studies, menopausal status in our analysis was strongly associated with hyper-LDL-cholesterolemia and overall dyslipidemia. Importantly, formal interaction testing demonstrated statistically significant effect modification by menopausal status only for hypo-HDL-cholesterolemia, suggesting that menopausal hormonal changes may differentially influence HDL metabolism in relation to sleep duration. However, associations between sleep duration and other lipid outcomes appeared broadly consistent across menopausal groups.

Several physiological mechanisms may explain the observed association between sleep duration and triglyceride levels. Sleep restriction has been linked to dysregulation of appetite-related hormones, including decreased leptin (an appetite suppressant) and increased ghrelin (an appetite stimulant), which promote increased caloric intake and weight gain [24–26]. Sleep disturbances are also associated with sympathetic nervous system activation, circadian rhythm disruption, and insulin resistance, all of which contribute to hepatic very-low-density lipoprotein production and impaired triglyceride clearance [27–29]. Triglycerides are particularly sensitive to short-term behavioral and hormonal changes, whereas LDL-C and HDL-C reflect longer-term lipid homeostasis influenced by genetic predisposition, chronic dietary patterns, and pharmacologic treatment [30, 31]. This metabolic distinction may explain why sleep duration demonstrated stronger associations with triglyceride levels than with LDL-C or HDL-C.

This study has several strengths. First, KNHANES employs a nationally representative multistage sampling design, allowing findings to be generalizable to Korean women in midlife. The use of standardized laboratory measurements and a large sample size further strengthen the internal validity and statistical power of the study. Second, the study incorporated menopausal status as a key biological variable and conducted stratified analyses, allowing us to explore potential heterogeneity in the associations between sleep duration and lipid profiles across reproductive stages. Third, we applied multiple complementary analytic approaches to strengthen the robustness of the findings. In addition to categorical regression models, we examined sleep duration as a continuous exposure using parametric linear and quadratic terms to explore potential functional forms of association. Sensitivity analyses were also conducted by excluding implausible sleep duration values and trimming extreme triglyceride values, and the results remained materially unchanged. Finally, formal interaction testing was conducted to evaluate effect modification by menopausal status, enhancing the methodological rigor of subgroup interpretations and reducing the risk of misinterpretation based solely on stratified estimates.

Several limitations should be considered when interpreting the findings of this study. First, the cross-sectional design precludes causal inference and raises the possibility of reverse causation. Metabolic disorders associated with dyslipidemia may themselves contribute to sleep disturbances through mechanisms such as sleep-disordered breathing, inflammation, and hormonal dysregulation [27, 32]. Therefore, longitudinal cohort studies and intervention trials are needed to clarify the temporal and causal relationships between sleep duration and lipid metabolism. Second, sleep duration was self-reported, which may introduce recall bias and measurement error. However, such misclassification is likely non-differential and would bias associations toward the null, potentially underestimating true relationships. Third, residual confounding cannot be excluded. Certain sleep-related factors, including sleep quality, circadian rhythm disruption, shift work, and sleep medication use, were not consistently available in KNHANES. Additionally, comprehensive dietary pattern measures and family history of dyslipidemia were not uniformly assessed across survey cycles. The absence of these variables may have resulted in incomplete adjustment for lifestyle and genetic influences on lipid metabolism. Fourth, menopausal status was based on self-reported menstrual history without hormonal confirmation such as FSH (follicle-stimulating hormone), which may have resulted in misclassification, particularly among perimenopausal women. Future studies incorporating hormonal measurements and explicitly examining perimenopausal women as a distinct group would provide more precise characterization of reproductive stage-specific associations. Fifth, body mass index (BMI) was included as a covariate in multivariable models. Because BMI may act as both a confounder and an intermediate variable, adjustment may attenuate observed associations. However, the persistence of the association with hypertriglyceridemia after full adjustment supports the robustness of this finding. Sixth, menopausal hormone therapy could influence lipid metabolism, but detailed information on hormone therapy was not available in KNHANES, limiting our ability to evaluate its potential impact [33]. Finally, the study population was limited to women aged 40–64 years, restricting generalizability to older populations. Further studies in broader age groups and diverse populations are warranted. Given that KNHANES employs a nationally representative sampling design, our findings are generalizable to Korean women aged 40–64 years during the study period. However, generalizability to other populations may be limited by differences in genetic background, lifestyle factors, and healthcare systems.

Cardiovascular disease remains a leading global cause of mortality, and dyslipidemia is frequently underdiagnosed and undertreated [1, 4]. In modern societies, sleep duration is increasingly disrupted by social and occupational demands. Our findings highlight an important but often overlooked connection between sleep hygiene and metabolic health. The observed associations between short and long sleep duration and hypertriglyceridemia suggest that sleep duration may represent a modifiable behavioral factor influencing triglyceride metabolism. These findings underscore the importance of considering sleep duration as part of comprehensive metabolic risk assessment and highlight the need for longitudinal studies to further clarify casual relationships.

## Conclusions

This study identified significant associations between sleep duration and hypertriglyceridemia among midlife women. Menopausal status was also associated with dyslipidemia, and interaction analyses suggested potential differences in sleep related associations for HDL-C outcomes. These findings highlight sleep duration as a potentially relevant behavioral factor associated with lipid abnormalities in midlife women. Further longitudinal studies are needed to clarify temporal relationships and underlying mechanisms.

Abbreviations: LDL, low-density lipoprotein; HDL, high-density lipoprotein; SD, standard deviation.

## Supplementary Information

Supplementary Material 1.

Supplementary Material 2.

## Data Availability

The data used in this study were obtained from the Korea National Health and Nutrition Examination Survey (KNHANES), conducted by the Korea Disease Control and Prevention Agency (KDCA). Most KNHANES datasets are publicly available on the official website ( https://knhanes.kdca.go.kr/ ); however, access to certain datasets requires KDCA approval. Researchers can request access by following the procedures outlined on the KNHANES website. The datasets used and/or analyzed in this study are available from the corresponding author upon reasonable request.
