# Supplementary figures and images for: Association between sleep duration and dyslipidemia in premenopausal and postmenopausal women
Source: BMC Public Health. 2026 Mar 17;26:1345. doi: 10.1186/s12889-026-27011-1 (PMC13107795; doi:10.1186/s12889-026-27011-1)

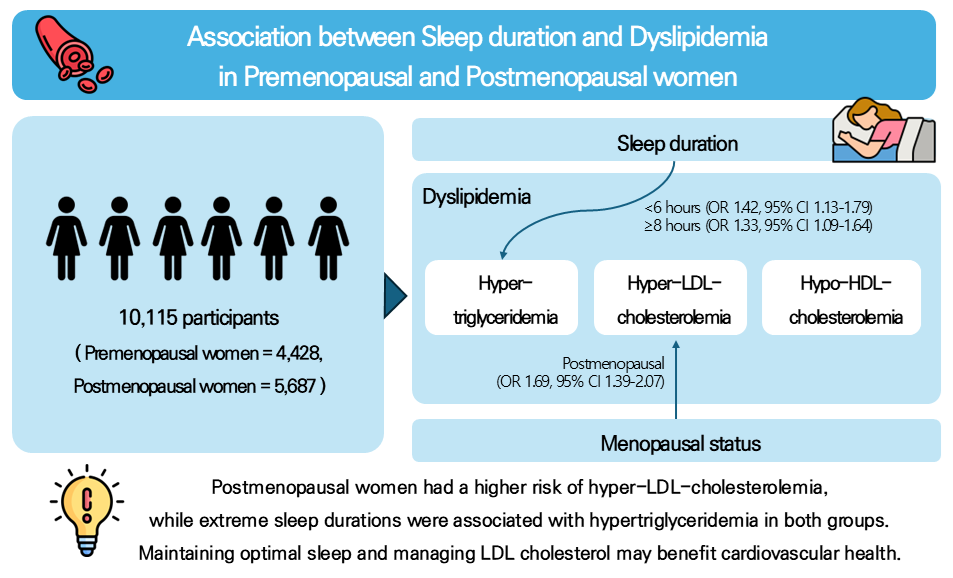

Supplement: Supplementary file 1 — Supplementary Material 1. [file 12889_2026_27011_MOESM1_ESM.png]
