# Supplementary material for: Association between sleep duration and dyslipidemia in premenopausal and postmenopausal women
Source: BMC Public Health. 2026 Mar 17;26:1345. doi: 10.1186/s12889-026-27011-1 (PMC13107795; doi:10.1186/s12889-026-27011-1)
Supplement: Supplementary file 2 — Supplementary Material 2. [file 12889_2026_27011_MOESM2_ESM.docx]

**Supplementary Information**

Continuous Modeling of Sleep duration

When sleep duration was modeled as a continuous variable in fully adjusted logistic regression models, neither linear term nor quadratic term was statistically significant for dyslipidemia or any lipid outcome (Table S1). These findings suggest that a simple parametric polynomial function may not adequately capture the relationship between sleep duration and lipid abnormalities. This supports the use of clinically meaningful categorical sleep duration groups in the main analysis, which allows for a more flexible assessment of potential non-linear associations.

To further evaluate potential non-linearity, sleep duration was modeled using both a linear term (Model 1) and combined linear and quadratic terms (Model 2). The quadratic term was not statistically for any outcome, indicating limited evidence of non-linearity under this parametric specification. Sleep duration was mean-centered prior to inclusion of the quadratic term to reduce multicollinearity and improve interpretability.

**Table S1.** Association between Sleep duration and dyslipidemia using linear and quadratic terms (fully adjusted models)

| Variables | Model | Sleep duration  (linear) | Sleep duration²  (quadratic) |
| --- | --- | --- | --- |
| Dyslipidemia | Model 1 ^a^ | 1.02 (0.98-1.06) |  |
|  | Model 2 ^b^ | 1.02 (0.98-1.06) | 1.00 (0.98-1.01) |
| Hyper-LDL-cholesterolemia | Model 1 | 1.00 (0.96-1.05) |  |
|  | Model 2 | 1.00 (0.96-1.05) | 1.00 (0.98-1.02) |
| Hyper-triglyceridemia | Model 1 | 0.99 (0.93-1.05) |  |
|  | Model 2 | 0.99 (0.93-1.05) | 1.01 (0.99-1.02) |
| Hypo-HDL-cholesterolemia | Model 1 | 1.02 (0.98-1.06) |  |
|  | Model 2 | 1.02 (0.98-1.06) | 1.00 (0.99-1.02) |

Footnote: OR = Odds Ratio; CI = Confidence Interval.

^a^ Model 1 includes the linear term for sleep duration only.

^b^ Model 2 includes both linear and quadratic terms for sleep duration.

All models were adjusted for menopausal status, age, economic activity, income quartiles, education level, hypertension, diabetes mellitus, smoking, drinking, physical activity, and BMI.

Sleep duration was mean-centered prior to inclusion of the quadratic term to reduce multicollinearity.

Sensitivity analysis

This study evaluated model fit by applying the Nagelkerke R² goodness-of-fit test and analyzing the area under the receiver operating characteristic curve (AUC), while accounting for the complex sampling design of the data (Table S2). Model 2 consistently demonstrated the highest Nagelkerke R² and AUC values, indicating superior model fit and predictive performance after adjustment for demographic, behavioral, and clinical covariates.

In addition, sensitivity analyses were conducted to assess the potential influence of extreme values. Triglyceride levels exhibited a highly right-skewed distribution (minimum 18 mg/dL, maximum 1951 mg/dL), and self-reported sleep duration ranged from 2 to 17.4 hours per day. Based on clinical plausibility, sleep duration <3 hours or >14 hours were considered implausible and excluded. Furthermore, the upper and lower 1% of triglyceride values were trimmed to reduce the potential impact of extreme observations. After applying these exclusions, the association between sleep duration and hypertriglyceridemia remained statistically significant, and effect estimates were materially unchanged compared with the main analysis (Supplementary Table S3).

**Table S2**. Model Performance for the Associations Between Sleep duration and Dyslipidemia using Multivariable Logistic Regression

| Variable | Crude | | Model 1 ^a^ | | | Model 2 ^b^ | | |  |
| --- | --- | --- | --- | --- | --- | --- | --- | --- | --- |
|  | Nagelkerke R^2^ | AUC (95% CI) | | Nagelkerke R^2^ | AUC (95% CI) | | Nagelkerke R^2^ | AUC (95% CI) | |
| Dyslipidemia | 0.002 | 0.52(0.51-0.53) | | 0.075 | 0.63 (0.62-0.64) | | 0.195 | 0.72 (0.71-0.73) | |
| Hyper-LDL-cholesterolemia | 0.001 | 0.51(0.50-0.53) | | 0.109 | 0.65 (0.64-0.66) | | 0.193 | 0.74 (0.73-0.75) | |
| Hyper-triglyceridemia | 0.007 | 0.54(0.52-0.56) | | 0.018 | 0.58 (0.56-0.60) | | 0.085 | 0.68 (0.66-0.70) | |
| Hypo-HDL-cholesterolemia | 0.000 | 0.51(0.50-0.52) | | 0.010 | 0.68 (0.66-0.70) | | 0.090 | 0.66 (0.65-0.67) | |

Footnote: AUC = Area Under the Curve; CI = Confidence Interval.

^a^ Model 1 adjusted for menopausal status

^b^ Model 2 additionally adjusted for age, economic activity, income quartiles, education level, hypertension, diabetes mellitus, smoking, drinking, physical activity, and BMI.

**Table S3.** Sensitivity analysis excluding extreme values for the association between sleep duration and hypertriglyceridemia (fully adjusted model)

|  | Main analysis OR (95% CI) | Sensitivity analysis OR (95% CI) |
| --- | --- | --- |
| *Sleep duration(h) (ref. 7-<8)* |  |  |
| <6 | **1.42 (1.13-1.79)** | **1.45 (1.14-1.85)** |
| 6-<7 | 1.10 (0.90-1.34) | 1.16 (0.94-1.43) |
| ≥8 | **1.33 (1.09-1.64)** | **1.32 (1.06-1.63)** |

Footnote: OR = Odds Ratio; CI = Confidence Interval.

Models were adjusted for menopausal status, age, economic activity, income quartiles, education level, hypertension, diabetes mellitus, smoking, drinking, physical activity, and BMI. (same covariates as Model 2 in Table 2, 3).
